# Editorial: Recent advances in diagnosis and treatment of brain tumors: from pediatrics to adults

**DOI:** 10.3389/fneur.2025.1606149

**Published:** 2025-05-27

**Authors:** John Bianco, Dimitrios N. Kanakis, Archya Dasgupta, Majaz Moonis, Cesare Zoia

**Affiliations:** ^1^Image Sciences Institute, University Medical Center Utrecht, Utrecht, Netherlands; ^2^School of Medicine, University of Nicosia, Nicosia, Cyprus; ^3^Advanced Centre for Treatment, Research and Education in Cancer (ACTREC), Mumbai, India; ^4^UMass Memorial Medical Center, Worcester, MA, United States; ^5^Unit of Neurosurgery, Department of Surgical Sciences, San Matteo Hospital Foundation (IRCCS), Pavia, Italy

**Keywords:** brain tumor, neuro oncology, pediatric neurosurgery, diagnosis, treatment

## Introduction

In the last few years, an impressive development has been achieved in the arena of new diagnostic and therapeutic approaches for brain tumors, both in children and adults. This has in turn led to the recognition of new tumor entities as well as to better categorization of the existing ones. The recent WHO classification of the CNS tumors (2021) has been entirely revised, and the term “integrated diagnosis” has since been applied, which refers to a combination of the classical histopathological diagnosis with the accompanying molecular results of some of the most common tumors. In addition, further progress has been made in the field of imaging, with the invention of more accurate methods and the improvement of previously established diagnostic modalities. As a result of the aforementioned achievements in the diagnosis of brain tumors, new treatment options have been introduced, cultivating in improved therapeutic response in several tumor entities.

Due to the immense progress not only in the field of diagnosis but also in the treatment of brain tumors, it is of utmost importance to present newly identified biomarkers and innovative techniques, which allow on the one hand more accurate diagnoses and on the other hand more precise therapeutic interventions. Since much research is conducted at present with this regard, the result of these investigations should be shared with the broader research community, in order to improve thereafter both the available diagnostic methods in the fields of histopathology and imaging and the therapeutic techniques in the areas of neurosurgery and chemo-/radio-therapy. Undoubtfully, this will contribute to accomplishing the target of “personalized medicine” in the public. This Research Topic assembles 23 contributions—spanning original research, reviews, case reports, methods, and clinical trials—to illuminate the multidisciplinary frontiers of brain tumor science, bring together new discoveries in the diagnosis and therapy of pediatric and adult brain tumors, where the evolving landscape of glioma management continues to integrate innovative diagnostic tools and therapeutic strategies.

## Evaluating biomarkers distribution, surgical intervention, and magnetic resonance spectroscopic imaging in the prognosis, differentiation, and diagnosis of brain tumors

According to Hu and Zhang, advanced multiparametric MRI (DSC, DWI, DTI, MRS) had the potential to non-invasively predict Ki-67 labeling index, a marker of tumor proliferation, in a cohort of 109 glioma patient. Their model, combining rCBVmax, rCBFmax, rADCmin, rFAmax, and Cho/Cr ratio, achieved high accuracy (*R*^2^ = 0.80), correlating with tumor grade. This approach could enhance preoperative planning by identifying high-proliferation gliomas, predicting prognosis before surgery, though validation in larger cohorts is needed. In another study by Yang et al., the cost effective diagnostic potential of peripheral blood parameters, including neutrophil-lymphocyte ratio (NLR), systemic immune-inflammation index (SII), and pan-immune-inflammation value (PIV), where gliomas could be distinguished from benign tumors, underscores their role in malignancy assessment. Supplementing these two studies, Lange et al. identified a 7-gene glutamatergic panel differentiating glioblastomas (GBMs) from brain metastases with 88% accuracy. Although larger validation is needed, this tool could supplement pathology in ambiguous cases, reducing reliance on invasive biopsies. Guo et al. developed a scoring system predicting ventriculoperitoneal shunt need post-pediatric tumor resection. They show that factors such as age (< 3 years), midline location, preoperative hydrocephalus, and total resection stratify risk, aiding postoperative monitoring, providing a practical evaluation. Scores ranging from 6 to 14 points indicate high risk, while the model also emphasizes blood loss as a novel, objective predictor linked to inflammation and CSF dynamics. The grim prognosis of diffuse intrinsic pontine gliomas (DIPGs), nowadays referred to as diffuse midline gliomas (DMGs) was reinforced by Boukaka et al., showing that benign brainstem tumors treated surgically display a survival rate of over 90%, compared to 3-year survival of just 2% for diffuse pontine gliomas. While stereotactic biopsy is not part of the standard of care in DMG of the pons, the heterogeneity of this disease advocated their use in providing critical on molecular and genetic characteristics that can guide treatment decisions, including entry into clinical trials. They advocate for individualized treatments based on molecular profiling to guide emerging therapies, stressing the urgency for targeted drug development, and highlighting the balance between aggressive resection (for benign lesions) and quality of life, and more biomolecular and genetic research for DMG. Future efforts must prioritize validating these tools in diverse cohorts and integrating molecular data into clinical algorithms, ensuring precision medicine becomes a tangible reality for glioma patients.

## Clinical trials assessing feasibility, efficacy and benefit of therapies in glioblastoma and craniopharyngioma

Two clinical trials presented in this Research Topic explore adjuvant strategies in glioma treatment, addressing duration of standard therapy and novel drug repurposing. Anvari et al. challenges the utility of extended temozolomide dosing (12 vs. 6 cycles) in high-grade gliomas. Despite comparable survival rates, the authors demonstrate that extended therapy showed no survival benefit, as well as lower completion rates, where toxicity, cost, and potential reduced salvage response underscore 6 cycles as standard. However, molecularly defined subgroups may warrant tailored approaches, necessitating further study. Pace et al. investigate drug repurposing, where they evaluated chlorpromazine combined with temozolomide in unmethylated MGMT GBM. In a phase II clinical trial, a median progression free survival of 8 months was achieved (vs. historical 5 months), with an overall survival of 15 months, meeting primary endpoints. They also found that the safety profile of chlorpromazine was manageable, and its repurposing to disrupt neuron-GBM signaling and therapeutic resistance merits phase III evaluation. What these studies do is to highlight the need of optimizing existing protocols, and the importance of exploring repurposed drugs to overcome resistance in glioma therapy. Similarly, in their brief research report, Hedrich et al. retrospectively document the feasibility of intracystic treatment with peginterferon alfa-2a in five patients (4 patients < 12 years, 1 adult patient) with cystic craniopharyngioma, observing cyst reduction with minimal toxicity, while also reducing hospital visits. Although some challenges such as cyst leakage need to be addressed, this approach aligns with the paradigm of treating craniopharyngioma as a chronic condition, prioritizing quality of life over aggressive interventions.

## Rare cases of CNS tumors and the importance of molecular profiling

The diagnosis and management of central nervous system tumors presents unique challenges, frequently requiring integration of advanced molecular profiling and multidisciplinary collaboration. Recent case reports presented in this Research Topic highlight these complexities, offering insights into diagnostic pitfalls, molecular advancements, and therapeutic strategies. Liu et al. present a case of intraventricular Rosai-Dorfman disease (RDD), a rare histiocytic disorder, in a young patient with no recurrence at 10-year follow-up following resection. Six similar cases in the literature were reviewed and showed that, although treatment guidelines for RDD have not been established, individualized surgical interventions and vigilant postoperative monitoring offer a favorable prognosis for this rare condition. The authors highlight the importance of considering RDD, though rare, in the differential diagnosis of intraventricular masses in pediatric patients. Wu et al. present a case of pleomorphic xanthoastrocytoma (PXA, WHO 2) with an NTRK fusion and a CDKN2A deletion in a 2-year-old patient with spontaneous intracranial hemorrhage. This case is one of few pediatric PXA reports that offers molecular profiling and illustrates the role of genomic testing in identifying targetable alterations, while underscoring hemorrhage as a rare presentation of low-grade gliomas. A case of synchronous IDH-NOS grade II (frontal) and IDH-mutant grade IV (parietal) astrocytomas is presented by Jia et al., which challenges the norms of glioma progression through molecular analysis revelations of divergent clonal origins, where an EGFR amplification in the parietal lesion was observed. This case highlights the necessity of comprehensive molecular workup in multifocal gliomas to inform surgical and adjuvant strategies. Edelbach et al. presents a case of glioblastoma in the brainstem, the diagnosis of which relied on molecular profiling after inconclusive biopsy. Although radiotherapy with concomitant and adjuvant temozolomide improved symptoms, this report stresses the dismal prognosis of infratentorial GBM and highlights the challenges of managing diffuse primary pontine glioblastoma, stipulating the need for more effective treatment options for this rare subtype of GBM. In another study, the Todai OncoPanel was used to analyse recurrent meningioma, revealing NF2 loss, CDKN2A deletion, and subclonal TRAF7 mutations. Ohara et al. highlighted the limitations of current meningioma therapies by showing that although high-risk markers were identified with this panel, no actionable targets were found. This case study advocates for expanded molecular panels and trials targeting pathways like PI3K or CDK inhibitors. A case of polymorphous low-grade neuroepithelial tumor of the young (PLNTY), harboring a FGFR3-TACC3 fusion and a TERT promoter mutation, was presented by Golub et al. showing that this entity mimics high-grade glioma in histological and molecular features. This case emphasizes the need for attentive follow-up of low-grade lesions such as PLNTY and the diagnostic value of methylation profiling to help elucidate the role and timing of adjuvant treatment. Consoli et al. presents two GBM cases which were misdiagnosed as autoimmune encephalitis due to atypical MRI features and false-positive onconeural antibodies, but later confirmed as GBM following biopsies prompted by unresponsiveness to immunosuppression treatment. This report warns against overreliance on serological markers in atypical presentations and advocates early biopsy in ambiguous cases. These case studies highlight that there is a need to prioritize efforts in validating molecular biomarkers in clinical trials, to expand targeted therapies, and to refine guidelines for rare entities. As molecular diagnostics evolve, so too must therapeutic paradigms, ensuring precision medicine transcends common tumors to address the full spectrum of CNS malignancies. For both pediatric DIPG/DMG and adult GBM, the future hinges on bridging diagnostic accuracy with innovative treatments, ensuring aggressive interventions are balanced against quality of life and the promise of tailored therapies.

## Reviewing technological advancements and patient centricity in diagnosing and treating CNS malignancies

The evolving landscape of neuro-oncology demands innovative diagnostic and therapeutic strategies, particularly for rare or complex CNS tumors. This Research Topic also contains a number of reviews that shed light on critical advancements, highlighting that improved outcomes can be achieved by integrating technology, molecular insights, and patient-centered care. Through a bibliometric analysis of 179 studies from the Web of Science core database, Abudueryimu et al. reveal the pivotal role of MRI surgical planning and recurrence monitoring following spinal schwannoma diagnosis, while highlighting disparities in terms of research quality between Eastern and Western institutions. They point out that while China leads in publication volume, institutes in Europe and America dominate in citation impact. To bridge quantity and quality, future efforts must prioritize standardized imaging protocols and translational studies to refine feature analysis, enhancement studies, and quantitative assessments. Progress in these domains raises the bar for diagnostic and therapeutic approaches for spinal intradural schwannomas, improving patient care and outcomes. Yu et al. performed a meta-analysis comparing [^1^8F]FET and [^1^8F]FDOPA PET for glioma recurrence diagnosis. They found that while both demonstrate similar specificity, [^1^8F]FDOPA shows superior sensitivity, attributed to its dual targeting of dopamine pathways and amino acid metabolism, although sample sizes were limited. As limited availability and higher costs hinder widespread adoption of [^1^8F]FDOPA, the authors advocate using hybrid approaches combining PET's molecular sensitivity with MRI's anatomical specificity. A narrative review by Chen et al. explores the evolving landscape of treatment efficacy of the complex intracranial tumor adamantinomatous craniopharyngioma. The take home message of this review is that alternative approaches for sustained disease control, such as subtotal resection paired with radiotherapy, which achieves comparable tumor control with fewer complications, could pose a paradigm shift away from radical resection, while emerging targeted therapies and cyst-directed treatments offer promise. Namiot et al. investigated brain tumor diagnosis by exploring the potential of *in situ* hybridization (ISH) techniques. By cross-referencing 513 records with the OMIM database, a large number of mutations suitable for ISH were pinpointed, such as amplifications in EGFR, MDM2, and MDM4, and deletions of PTEN, CDKN2/p16, TP53, and DMBT1 that correlate with poor prognosis in glioma patients, as well as other chromosomal anomalies across different non-glioma brain tumors. Though highlighting the potential of this technique in diagnosing and prognosticating various brain tumors, the authors concede that while ISH enhances subclassification, its inability to resolve small mutations limits standalone use, urging integration with next-generation sequencing for comprehensive profiling. Many therapeutics fail in the clinic because of the blood-brain barrier (BBB) disallowing chemo-/immune-therapies to reach the target site. Recent research analyzing the application of ultrasound for therapeutic purposes has highlighted the role of focused ultrasound (FUS) as a treatment modality for gliomas, as presented by Nwafor et al.. FUS has emerged as a dual tool for thermal ablation and blood-brain barrier disruption (BBBD), enhancing chemotherapeutic delivery beyond the BBB. While challenges remain and further investigation is still needed, early clinical trials show promising results in enhanced drug delivery in brain tumors using this non-invasive approach. The reviews outlined above map a path forward where technology and patient-centricity converge, urging clinicians and researchers to embrace multidisciplinary collaboration for transformative progress in neuro-oncology.

## Liquid biopsies and artificial intelligence for identifying tumor burden and monitoring progression

Technological advancements that promise to refine diagnostics, enhance treatment efficacy, and support clinical decision-making continue to evolve, where novel strategies such as liquid biopsies and artificial intelligence (AI) integration underline the field's trajectory toward precision medicine. Barber et al. investigated techniques for enriching circulating tumor cells (CTCs) by comparing four CTC enrichment methods to address GBM's diagnostic challenges. The authors found that the ScreenCell^®^ system emerged as the most viable for clinical use due to its simplicity, speed, and biomarker-agnostic approach, achieving CTC isolation via size-based filtration, with minimal cell loss, while having compatibility with downstream analysis. Dheepak et al. introduce a novel imaging framework that integrates Gray-Level Co-occurrence Matrix (GLCM) and Local Binary Pattern (LBP) features, augmented by interaction features derived from their outer product. By using this approach in classifying gliomas, meningiomas, and pituitary tumors using a linear SVM classifier, they were able to achieve an accuracy rate of 98.84%. These methods have the potential to improve the precision of medical image processing significantly, in turn assisting clinicians to provide more accurate diagnoses and treatments for brain tumors. Along these lines, Mut et al. explored the role of AI in GBM surgery, highlighting its strengths in tumor segmentation and resection extent prediction via radiomics and connectomics. However, the authors go on to report that predicting post postoperative outcomes was limited due to data variability and less quantifiable patient-related factors. As such, they advocate for standardized datasets, multimodal imaging integration, and ethical AI frameworks, and conclude that while AI can aid in training, it cannot yet replicate the nuanced judgment of experienced neurosurgeons.

## Conclusions and future perspectives

This Research Topic combines 23 studies spanning diagnostics, therapeutics, and emerging technologies in neuro-oncology, emphasizing the multidisciplinary advances in brain tumor diagnosis and therapy and progress toward precision medicine. Key advancements include non-invasive diagnostic tools that can predict glioma proliferation, differentiate gliomas from benign tumors, and provide molecular profiles that further refine diagnostics. Therapeutic innovations also challenge conventional protocols, questioning the benefit on overall survival of extended treatment in patients with newly diagnosed GBM, advocating for molecularly tailored approaches. Repurposing of drugs was also shown to hold promise by demonstrating improved progression free survival in unmethylated MGMT GBM, warranting further phase III trials. For rare tumors, adapted treatment protocols, prioritizing quality of life over aggressive surgery, has been shown to be effective and well tolerated. The complexity of diagnosis, where different entities can give rise to ambiguities, shows the need for advancing genomic testing and molecular profiling, where imaging, liquid biopsies and artificial intelligence have emerged as potentially transformative tools for tumor classification and intraoperative decision-making. Drug delivery enhancement through FUS mediated BBB-opening could increase efficacy, reduce toxicity, and improve overall quality of life. These studies collectively promote the shift toward minimally invasive diagnostics, targeted therapies, and data-driven tools, where molecular insights can be integrated with multidisciplinary care, balancing aggressive intervention with patient-centered outcomes. While challenges such as validation requirements, lack of druggable targets for rare subtypes, as well as cost barriers and availability remain, technological breakthroughs in neuro-oncology as outlined in this Research Topic can bridge the gap between innovation and impactful outcomes, delivering innovation and cutting-edge therapies that bring the field closer to personalized medicine for both common and rare CNS malignancies.

